# Investigation of Inversion Polymorphisms in the Human Genome Using Principal Components Analysis

**DOI:** 10.1371/journal.pone.0040224

**Published:** 2012-07-09

**Authors:** Jianzhong Ma, Christopher I. Amos

**Affiliations:** Department of Genetics, The University of Texas M. D. Anderson Cancer Center, Houston, Texas, United States of America; Queen’s University Belfast, United Kingdom

## Abstract

Despite the significant advances made over the last few years in mapping inversions with the advent of paired-end sequencing approaches, our understanding of the prevalence and spectrum of inversions in the human genome has lagged behind other types of structural variants, mainly due to the lack of a cost-efficient method applicable to large-scale samples. We propose a novel method based on principal components analysis (PCA) to characterize inversion polymorphisms using high-density SNP genotype data. Our method applies to non-recurrent inversions for which recombination between the inverted and non-inverted segments in inversion heterozygotes is suppressed due to the loss of unbalanced gametes. Inside such an inversion region, an effect similar to population substructure is thus created: two distinct “populations” of inversion homozygotes of different orientations and their 1∶1 admixture, namely the inversion heterozygotes. This kind of substructure can be readily detected by performing PCA locally in the inversion regions. Using simulations, we demonstrated that the proposed method can be used to detect and genotype inversion polymorphisms using unphased genotype data. We applied our method to the phase III HapMap data and inferred the inversion genotypes of known inversion polymorphisms at 8p23.1 and 17q21.31. These inversion genotypes were validated by comparing with literature results and by checking Mendelian consistency using the family data whenever available. Based on the PCA-approach, we also performed a preliminary genome-wide scan for inversions using the HapMap data, which resulted in 2040 candidate inversions, 169 of which overlapped with previously reported inversions. Our method can be readily applied to the abundant SNP data, and is expected to play an important role in developing human genome maps of inversions and exploring associations between inversions and susceptibility of diseases.

## Introduction

Common structural variations in the human genome such as deletions, duplications, and inversions are known to be associated with disease susceptibility [1]–[3] and to be subject to selection [4]. Among different types of structural variations, characterization of inversions in the human genome remains a difficult, or at least laborious, task because of the lack of a high-throughput technique for detecting them. Traditionally, standard cytogenetic approaches, such as fluorescence in situ hybridization (FISH)-based assays, are used to detect an inversion. Only recently, using fosmid cloning and paired-end sequencing, have studies been successful in genome-wide mapping of inversion breakpoints [5], [6]. Currently, 953 inversion regions are listed in the Database of Genomic Variants (DGV) [7]. However, since many of these overlap and may actually represent the same locus, there are a total of 517 unique autosomal inversion locations in the database. Although the sequencing-based method has been successfully used to screen for inversions, it has some limitations [8] and does not efficiently apply to large number of samples that are needed to characterize inversions in a population and detect their association with diseases.

A cost-efficient way of detecting and characterizing inversions may be based on the widely available high-density genotype data of Single Nucleotide Polymorphisms (SNPs). Unlike other types of structural variations such as deletions that cause miscalled genotypes, in an inverted segment of chromosome, the allele density is unaltered and the alleles are mapped to their locations on the reference genome rather than the physical locations. Nevertheless, effects of inversion variations are still manifested in the statistical properties of the SNP genotypes. Because the physical ordering and mapped ordering of the SNPs are different, one often observes an unusually higher level of long-range linkage disequilibrium (LD) and an unusually lower level of short-range LD [9]. An LD-based inversion statistic has been developed by Bansal et al. to detect inversions from the International HapMap data [10]. This approach, however, has little power when the inversion frequency is lower than 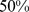 in a population. Another approach, by Sindi et al., is based on a probabilistic model of haplotype frequencies around inversion breakpoints, and is shown to have higher power and can be used to estimate inversion frequencies [11]. A recent generalization of this method by Cáceres et al. [12], as implemented in an R package, inveRsion, has made it possible to identify novel inversions and infer inversion status from genotype data with high sensitivity. Very recently, a bioinformatics tool, based on multidimensional scaling, has been developed for genotyping of the 8p23 inversion using unphased SNP data [13]. In addition, SNPs that are tightly linked to an inverted region can serve as a surrogate marker for the inversion, e.g. for the 17q21.31 inversion [4]. For the inversion at chromosome 8p23.1, Bosch et al. have identified 16 SNPs in strong LD with this inversion, and have used these SNPs to indirectly infer the inversion genotypes of some HapMap samples [14].

In this paper, we propose a novel statistical method for detecting and characterizing inversions from high-density SNP genotype data. Our method is based on principal components analysis (PCA), which has recently been widely used in population genetics and genetic epidemiology to detect population structures and correct for bias caused by population stratification in genetic association tests. Our rationale for applying PCA in the study of inversion polymorphisms is as follows. For an inversion polymorphism, if recombination is suppressed between the inverted and non-inverted segments [15]–[20], these two segments of different orientations in the local region represent two distinct lineages that have been diverging for many generations and accumulating mutations independently. SNPs within the inverted region should therefore have different statistical properties, as if they were from different populations. Using genotype data in the inversion region, individuals can be classified as different “populations” according to their inversion genotypes, and this population structure can be readily detected using PCA. Specifically, the inversion heterozygous individuals can be viewed as a perfect 1∶1 admixture of the two types of inversion homozygous populations, resulting in a special pattern in the distribution of samples in the space spanned by the first few eigenvectors. This special pattern, consisting of three equidistant stripes, as demonstrated using simulated data, is indicative of inversions and can be used to infer the inversion status of the samples. Here, we have applied our method to the genotype data from Phase III of the International HapMap project [21] and genotyped the samples for known or predicted inversion polymorphisms, including the two well-known inversions at 8p23.1 and 17q21.31. We have also performed a genome-wide scan for inversion polymorphisms based on our proposed method, and this generated 2040 candidate inversions in the HapMap populations. Some of these correspond to about one-third of the 517 known inversions listed in the DGV. Many of the rest may represent true inversions and thus deserve investigation and validation using sequencing-based and cytogenetic approaches. Our method can be used to infer inversion genotypes from GWAS data, which is widely available, and thus will find increasing use in exploring the influence of inversions in the development of diseases.

## Results

### Inversion as a Special Admixture

**Figure 1:**
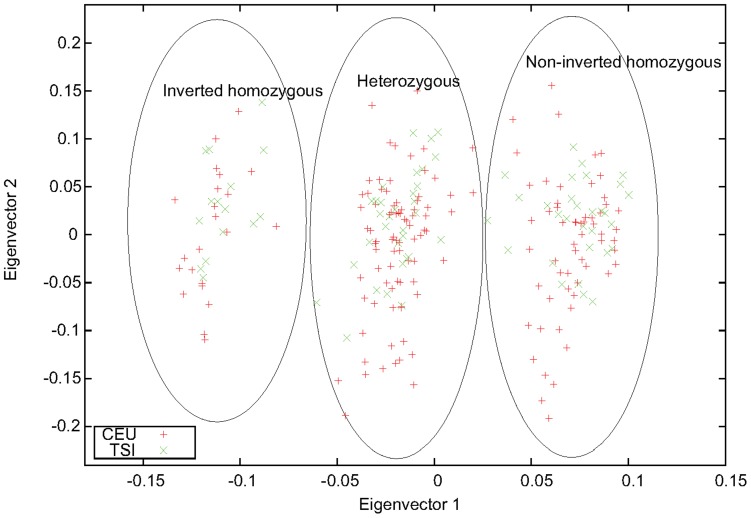
The three-stripe pattern as a manifestation of inversion. The first two eigenvectors obtained from PCA performed for the two Caucasian HapMap populations, CEU and TSI, using markers inside the 8p23.1 inversion region. The inversion genotypes were inferred by combining this figure with results from other PCA (see text for details).

A well-known inversion polymorphism on chromosome 8p23.1 (chr8∶7225962-12487029, hg18) has been found to be manifested in the pattern of the PC scatter plot (PC-plot) [22]. Along the second-largest PC, samples from Caucasians are distributed in three equidistant clusters when PCA is performed using genome-wide SNP data. We observed the same pattern in the space spanned by the first two eigenvectors for our PCA with individuals treated as features [23] performed for the two Caucasian populations of the HapMap project, CEU and TSI (data not shown). If only markers inside the inversion region are used for PCA, as shown in Figure 1, variations caused by the inversion polymorphism will dominate and the three clusters will be distributed along the first eigenvector. This three-stripe pattern has been attributed to LD caused by inversion [22].

However, in our previous study of applications of PCA on detecting population structure, we noticed that this kind of pattern actually reflects the fact that samples in the middle cluster are admixtures of those located in the two side clusters, as demonstrated by simulations [23]. The location of the cluster of admixed samples is determined by the admixture proportion. When a third population is included in PCA, this pattern usually persists to two eigenvectors, leading to a two-dimensional clustered pattern discovered in [24]. Therefore, we propose that the three-stripe pattern in the inversion region is a special case of admixture: because of suppression of recombination in heterozygotes, the inverted and non-inverted segments act as if they were in different populations and are represented by the two side clusters, and the inversion heterozygotes are a perfect 1∶1 admixture of the two homozygotes and thus are represented by the middle cluster, which is in the middle of the two clusters.

**Figure 2:**
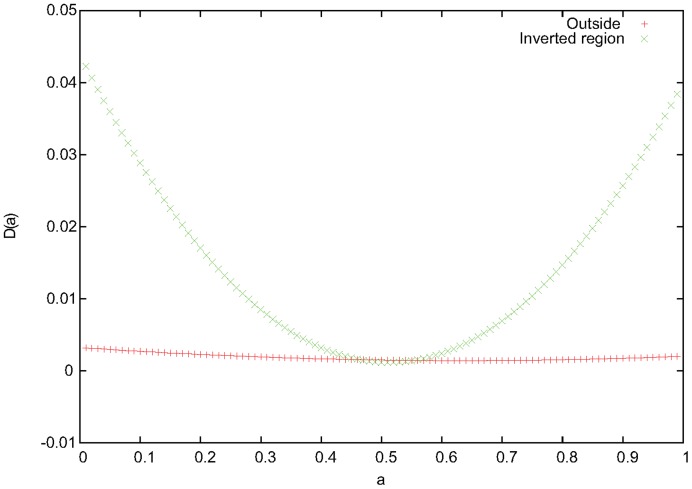
Analysis of allele frequency for SNPs inside and outside the 8p23.1 inversion region. The variance of 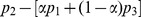, Δ(*α*), for different values of the admixture proportion 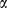 inside and outside the 8p23.1 inversion region for CEU and TSI. The results imply that, inside the inversion region, the samples in the middle stripe in Fig. 1 are admixtures of the samples in the two side stripes with an admixture proportion 0.5, whereas outside the inversion region, the three stripes are single populations.

In Figure 1, grouping according to the inversion genotypes, especially for those near the boundary between the heterozygotes and the non-inverted homozygotes, was based on analysis by combining results of PCA performed for different combinations of HapMap populations (Figures S1, S2, S3). Using this inversion genotyping information, we estimated the allele frequencies of the three clusters inside and outside of the inversion region. We found that, for markers inside the inversion region, the allele frequency of the middle cluster (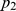) can be expressed in terms of those of the two side clusters as
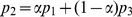
(1)only when 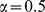. This is the same expression as for an admixed population of the two homozygotes with an admixture proportion 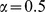. Outside the inversion region, the same expression is valid for any values of 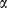 where 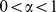, meaning that the three clusters are of the same population (Figure 2). We thus propose that the pattern of the eigenvector-plot or the PC-plot for PCA performed locally can be used to detect inversion polymorphisms and can be used to infer the inversion genotypes of individuals from high-density SNP genotype data. In the Methods section, we define a parameter, 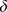, that goes to zero if the centroid of the middle cluster is located in the middle point between those of the two side clusters, reflecting the fact that 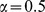. This parameter will be used to set a criterion in detecting inversion polymorphisms (see Methods).

### Simulation Results

**Figure 3:**
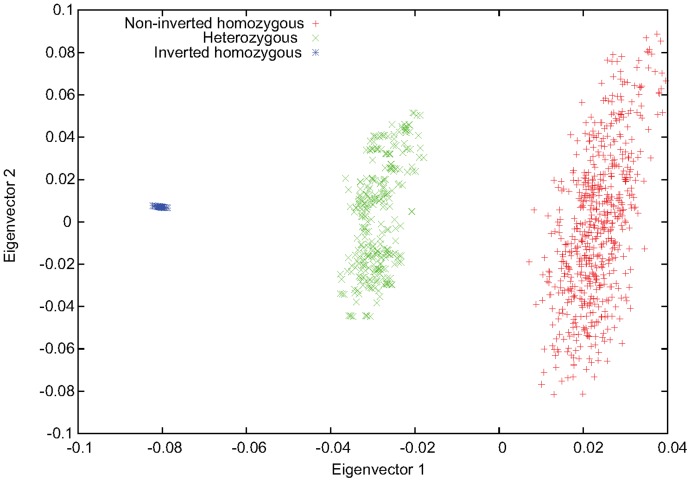
The three-stripe pattern caused by inversion for a typical simulated data set. The first two eigenvectors obtained from PCA performed for a simulated data set. The inversion genotypes represented by different colors were given by the simulating program, invertFREGENE.

We estimated the power of our method to detect inversions from SNP genotype data using simulated inversions generated by recently developed software, invertFREGENE [25]. For the simulating parameters, such as rates of recombination and mutations, we used the values tested in [25]. We simulated inversions of varying lengths (from 100 Kb to 2 Mb) and two different frequencies, 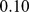 and 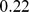. We sampled the genotypes for N = 1000 individuals and performed PCA inside the inversion region. A typical example of the simulated data sets for inversion frequency 0.22 and length 800 Kb is given in Figure 3, where a three-stripe pattern can be clearly seen for the eigenvector-plot. We created 100 replicates of data sets for each of the scenarios given by the inversion frequency and length by changing the random seed for recombination. For each of the replicates, if a three-stripe pattern can be identified in the eigenvector-plot according to the criteria set by the K-means clustering algorithm (see Methods for details), and the resulting inversion genotypes were consistent with the true inversion status obtained from the output of invertFREGENE, our method was considered successful. The power was measured by the fraction of replicates for which our method was successful. Results for all simulated scenarios are summarized in Table 1. The results indicate that the power of our method increases with increasing frequency of the inversion. When the frequency was close to 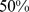, the power of our method was comparable to that of inveRsion [12] (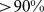) if the inversion region was not too short. Even for the relatively low frequency, 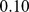, our method had significantly higher power (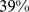) than the two statistical methods of Bansal et al. [10] and Sindi et al. [11] (see Fig. 2 in [10] and Fig. 3 in [11]). When the inversion region was too long (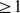 Mb), however, the power was reduced. We anticipate that, as the length of inversion increases, genetic variation among individuals of same orientations is getting larger than that between the two orientations, leading to a less clear three-stripe pattern.

**Table 1:** Power of the PCA-based approach to detect simulated inversions.

Inversion frequency	100 Kb	350 Kb	800 Kb	1 Mb	2 Mb
	inversion	inversion	inversion	inversion	inversion
0.10	0.23	0.39	0.49	0.39	0.40
0.22	0.57	0.70	0.84	0.75	0.75
0.45	0.77	0.93	1.00	0.93	0.91

To estimate the false positive rate of our proposed method, we created 100 replicates of data sets for which no inversion was simulated in the region of interest and all other simulating parameters were kept unchanged. In nine of these replicates, we found a clear three-stripe pattern according to the same criteria used for estimating the power when markers inside the same region were used to perform PCA. Although we do not expect the simulated data to fully represent the true reality, our results implied that other mechanisms than inversions may also result in a three-stripe pattern. If a genome region includes only two major haplotypes for reasons other than inversions, a three-stripe pattern should be also be observed. Distinguishing between such kind of regions and inversion polymorphisms is beyond the capability of the PCA approach.

### The 8p23.1 Inversion Polymorphism

**Figure 4:**
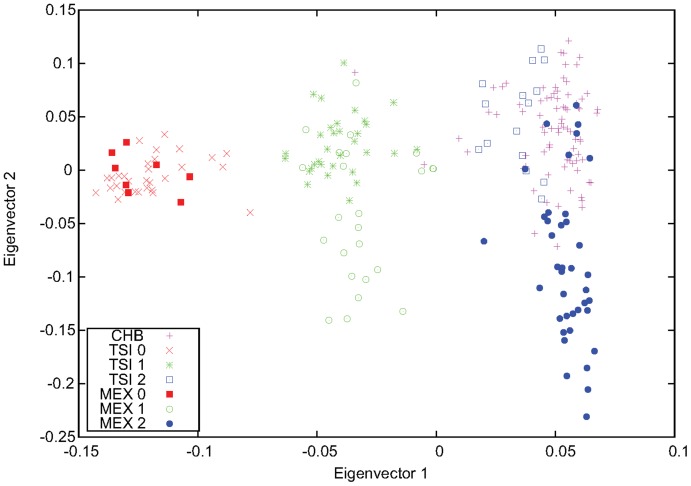
Genotyping of the 8p23.1 inversion for CHB using MEX and TSI. The first two eigenvectors obtained from PCA performed for CHB, MEX, and TSI using markers inside the 8p23.1 inversion region. Two individuals from CHB, NA18605 and NA18620, were identified as inversion heterozygous, and all other CHB samples were inverted homozygous.

To characterize the well-known 8p23.1 inversion polymorphism, we first performed PCA for each of the 11 HapMap populations (Figure S1). Except for MEX, TSI, and probably GIH, the three-stripe pattern could not be clearly seen, for different reasons. First, the sample sizes may not be large enough. Second, in some populations, such as the three Asian populations, this inversion is not polymorphic (namely, there are not variations for this inversion polymorphism). Finally, there existed unknown variations within this inversion region for some of the populations, such as CEU. We thus performed PCA for various different combinations of populations, mostly including MEX and TSI (Figures S2 and S3). Using Figure 1, the results from the single-populations PCA for TSI, MEX, and GIH (Figure S1), and the results for pooled data (Figures S2 and S3), we inferred the inversion genotypes of all individuals in the five populations (ASW, CEU, GIH, MEX, and TSI). The genotyping was accurate except for ASW, for which the boundaries between the stripes were not clear enough. The inversion genotypes of these populations are listed in Table S1. For the Asian populations, CHB, CHD, and JPT, we found that almost all of the samples are inversion homozygous, with two exceptions in CHB who seemed to be heterozygous (Figure 4 and Figure S2).

For the three African populations, none of them seemed to be mingled with any cluster of the three well-identified inversion genotypes of the Caucasians, but they all showed similarity with the non-inverted clusters (Figure S2). This may indicate that all of the African populations are of the original ancient type, but after a long period of evolution, the genetic variations within this wide inversion region are significantly different from those found in the non-inverted haplotypes in other populations. In Table 2, the genotype frequencies and allele frequencies of the 8p23.1 inversion calculated from the genotypes of inversion are listed for the non-African HapMap populations. Also listed in Table 2 are the P values of the HWE tests for this inversion. No deviation from HWE (P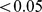) was observed for the inversion in any of the populations genotyped.

**Table 2:** Genotype and allele frequencies and the P values for HWE tests of the 8p23.1 inversion.

	Homozygous		Homozygous	Inversion	Hardy-Weinberg
Population	non-inverted	Heterozygous	inverted	frequency	P values
ASW	0.61(43)	0.31(22)	0.08(6)	0.24	0.2020
CEU	0.36(58)	0.51(82)	0.14(22)	0.39	0.5083
CHB	0.00(0)	0.02(2)	0.98(80)	0.99	1.0000
CHD	0.00(0)	0.00(0)	1.00(70)	1.00	1.0000
GIH	0.14(12)	0.54(45)	0.31(26)	0.58	0.3688
JPT	0.00(0)	0.00(0)	1.00(82)	1.00	1.0000
MEX	0.11(8)	0.34(24)	0.55(39)	0.72	0.2365
TSI	0.39(30)	0.42(32)	0.19(15)	0.40	0.2406

Determination of the inversion orientations of the two homozygous clusters was based on the results of PCA for CEU and TSI (Figure 1), which are genetically similar and have sufficient variations in terms of this inversion polymorphism. We assumed that the left stripe in Figure 1 corresponded to inverted homozygous because of its smaller variation compared with the other stripe along the second eigenvector. However, this is not always the case; sometimes the inverted homozygous cluster has a larger variation, for example, in the single-population PCA for MEX and TSI (Figure S1). In the case of MEX, the larger variation within the inverted homozygous cluster is due to its large sample size: 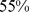 of MEX individuals are inverted homozygous.

As a validation of the PCA-based genotyping, Mendelian consistency was checked for populations with family information in which the 8p23.1 inversion is polymorphic: ASW, CEU, and MEX (Table S2). As shown in Table S2, segregation of this inversion in all these families followed the Mendelian law. Also, our genotyping results were consistent with those obtained using other approaches and validated by FISH for nine CEU samples given in [14], seven CEU samples given in [26], 63 CEU samples given in Table S1 in [13], and 20 CEU samples given in Table S3 in [12]. Specifically, our inversion genotypes for the two CEU samples (NA11831 and NA11840) were in agreement with the experimental results by FISH, while inveRsion gave different genotypes [12]. Note that the orientation of the inverted allele in Bosch et al. [14] and Deng et al. [26], which is the same as what we determined here, is referred to as non-inverted in Cáceres et al. [12] and in Salm et al. [13]. Our conclusion that almost all Asian individuals are inversion homozygous is in agreement with that of [26]. Specifically, the two inversion heterozygous individuals in the CHB population, NA18620 and NA18605, identified using our method (Table S1), were confirmed by FISH analysis [13], [26].

However, our results were different from those given by by Antonacci et al. [6] for two CHB samples (GM18529 and GM18571) and one JPT sample (GM18966), and those for some of the CHB and JPT samples listed in Table S1 in [13]. Moreover, for the Asian populations, our results (and those of [26]) for the inversion frequency are inconsistent with those obtained in [6], [14] and [27]. In [6] and [14], the inversion frequency are estimated as 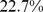 (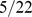 chromosomes) and 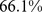 (for 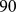 individuals), respectively, for the HapMap Asian populations. The study in [27] of 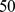 Japanese shows that the inversion frequency is 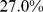. Bosch et al. [14] conjecture that the discrepancies among these studies may be due to mosaicism for the inversion resulting from mitotic recombination observed in their FISH analysis. Given that the results obtained in [6] and [27] are based on direct genotyping using cytogenetic analysis and are consistent with each other, we propose here another possible explanation for the discrepancy between these results and those obtained using statistical analysis given here and in [26] and [14] as follows. The inversion region at 8p23.1 is too large and thus recombination might be only moderately suppressed for the Asian populations, and thus the inferences from PCA are not reliable. However, it remains to explain why this is not the case for other populations, such as GIH and MEX, and why almost all Asian samples are genetically similar to the inversion homozygous individuals from other populations inside this inversion region. Finally, it is also possible that the PCA-based approaches failed to genotype this inversion correctly because it is recurrent in the Asian populations, as pointed out by Antonacci et al. [6].

### The 17q21.31 Inversion Polymorphism

**Figure 5:**
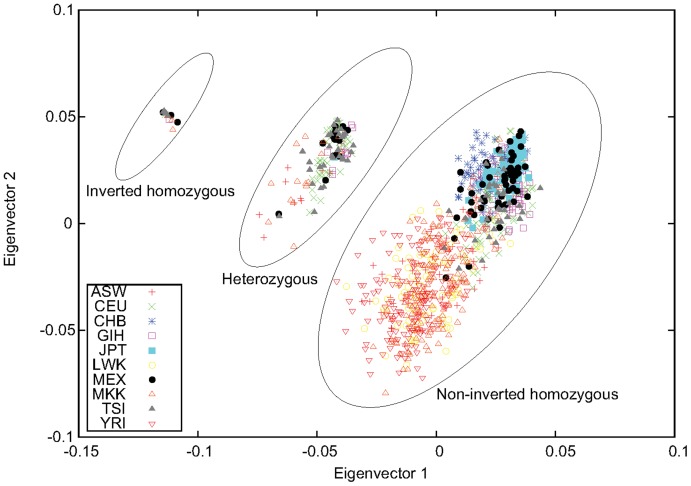
Genotyping of the 17q21.31 inversion for all HapMap populations except for CHD. The first two eigenvectors obtained from PCA performed for all HapMap populations except for CHD using markers inside the 17q21.31 inversion region.

**Figure 6:**
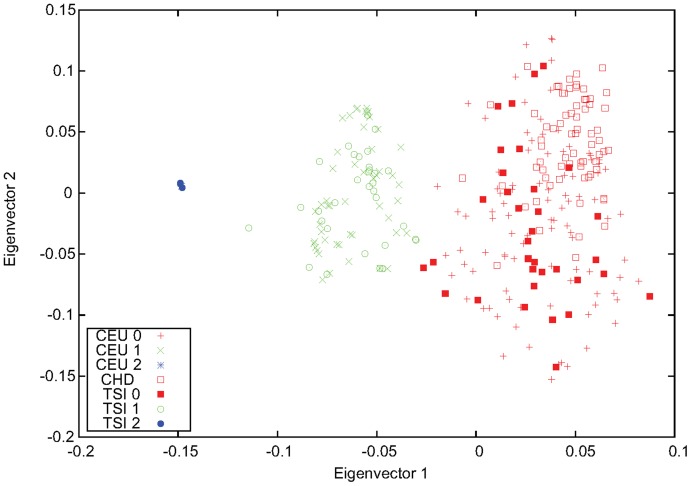
Genotyping of the 17q21.31 inversion for CHD using CEU and TSI. The first two eigenvectors obtained from PCA performed for CEU, CHD, and TSI using markers inside the 17q21.31 inversion region. All CHD samples were found to be non-inverted homozygous.

The 17q21.31 inversion (chr17∶40899921-41989253, hg18) is a recently discovered polymorphism [4]. Using all but one (CHD) HapMap populations, we observed a clear three-stripe pattern for the first two eigenvectors of PCA performed using the 140 common markers inside the 17q21.31 inversion region (Figure 5). To determine which side stripe corresponds to which homozygous inversion status, we suppose that the inverted segment originated from a single founder mutation and hence has very limited variations. Therefore, the shorter side stripe should be inverted homozygotes. When CHD was included in PCA, the pattern disappeared and no inversion could be detected. The reason is that the total number of common markers shared by all 11 populations was just 91, and probably most inversion-informative markers were not shared by all populations. When only CEU and TSI were combined with CHD for PCA with 116 common markers, the three-stripe pattern appeared and the samples of CHD were found to all be non-inverted homozygous (Figure 6). The other Chinese population, CHB, was found to be all non-inverted homozygous, as well. JPT was determined to be non-inverted homozygous for all but one individual, NA19085, who seemed to be heterozygous for this inversion polymorphism. It is interesting to note that MKK is the only African population in which the 17q21.31 inversion is polymorphic. This may imply that humans living on other continents share ancestry with MKK before dispersal out of Africa.

Results of PCA for each individual population are shown in Figure S4. Populations with all three inversion genotypes showed a clear three-stripe pattern, except for CEU, which showed additional structure as in the case of the 8p23.1 inversion. It should be noted that the stripes from PCA performed for a single population were perpendicular to the axis of the first eigenvector, whereas the stripes in Figure 5 for all populations were oblique. This reflects the fact that for a single population, variation was fully due to inversion and was hence completely addressed by the first eigenvector, whereas for the combined data, inter-population variations within each stripe were large enough to be addressed together with the inter-stripe variations by the first two eigenvectors.

Genotypes of all HapMap populations are given in Table S1. Mendelian consistency was confirmed using the genotypes for all populations with family information (Table S2). Table 3 shows the genotype frequencies, allele frequencies, and the P values of the HWE tests for the 17q21.31 inversion calculated from the estimated inversion genotypes for all HapMap populations. Except for CEU and MKK, no deviation from HWE (P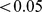) was observed for this inversion. In CEU, there is an excess of inversion heterozygotes. In MKK, however, the deviation from HWE is due to an excess of inverted homozygotes. We anticipate that these deviations from HWE may be due to the small sample sizes or to the relatedness between the parents and their children in these two populations.

**Table 3:** Genotype and allele frequencies and the P values for HWE tests of the 17q21.31 inversion.

	Homozygous		Homozygous	Inversion	Hardy-Weinberg
Population	non-inverted	Heterozygous	inverted	frequency	P values
ASW	0.85(60)	0.14(10)	0.01(1)	0.08	0.3973
CEU	0.60(98)	0.39(63)	0.01(1)	0.20	0.0058
CHB	1.00(82)	0.00(0)	0.00(0)	0.00	1.0000
CHD	1.00(70)	0.00(0)	0.00(0)	0.00	1.0000
GIH	0.82(68)	0.17(14)	0.01(1)	0.10	0.5501
JPT	0.99(81)	0.01(1)	0.00(0)	0.01	1.0000
LWK	1.00(83)	0.00(0)	0.00(0)	0.00	1.0000
MEX	0.73(52)	0.23(16)	0.04(3)	0.15	0.3507
MKK	0.88(151)	0.09(16)	0.02(4)	0.07	0.0044
TSI	0.44(34)	0.42(32)	0.14(11)	0.35	0.4567
YRI	1.00(163)	0.00(0)	0.00(0)	0.00	1.0000

Our genotyping results for the 17q21.31 inversion were in agreement with those assessed by FISH for the HapMap samples given in Table S2 in [6]. Our results were also in agreement with those for all 24 HapMap samples given by inveRsion listed in Table S2 in [12]. The genotype frequencies of this inversion for all 11 HapMap populations given in Table 3 were also consistent with the results obtained by using inveRsion in Table 3 in [12], except for small differences for ASW, CEU, and TSI.

### Potential Novel Inversion Polymorphisms

We conducted an autosomal genome-wide scan for inversion polymorphisms using the pooled data of the two Caucasian populations (CEU and TSI) by performing PCA within each window. Since the windows that satisfied the criteria WSS 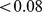 and 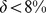 (see Methods) may overlap or be close to one another and thus might identify the same inversion region, we clustered the candidate windows if they overlapped or if the corresponding inversion genotypes overlapped by 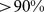. A candidate inversion was then defined as the whole region including all candidate windows in a cluster. Our scan yielded a total of 2040 candidate inversion polymorphisms (Table S3). Note that these predictions have to be taken with caution, because there are other biological processes (such as balancing selection) that can generate the observed three-stripe patterns detected by PCA. Since our simulations using invertFREGENE did not include this kind of biological processes, the false positive rate in inversion detection using real data might be higher than that estimated by our simulation (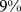).

Of the 2040 predicted inversions, 169 overlapped with the 517 non-redundant inversion polymorphisms listed in the DGV. The overlapping percentage of each of the 169 DGV inversions is defined as the length overlapping with one of the predicted inversions divided by its total length given in DGV, and is listed in Table S3. We can see that 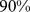 of the 169 inversions overlap with our predicted regions by at least 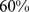, and 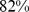 of them are completely covered by a predicted inversion region. Possible reasons why we were not able to detect the remaining 348 inversion polymorphisms listed in the DGV are as follows. First, many of the inversions may not be polymorphic in the Caucasian populations, and thus cannot be detected using the PCA approach. Second, our scanning method uses a fixed window size in terms of the number of markers and thus may miss some inversion regions that are too short or too long compared with the window we used. Finally, to control the false positive rate, we have used stringent criteria for identifying a novel inversion candidate. The criteria for WSS and 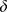 were set based the results of PCA for the 8p23.1 inversion polymorphism. The three-stripe pattern obtained for a window that is less obvious than the one in Figure 1 will not be selected as a candidate.

The remaining 1871 regions listed in Table S3 are the candidates for novel inversions we found using PCA. This is by no means a complete list, even for large inversions, within which there are enough markers for meaningful PCA. We expect that this number would increase significantly if other populations were used in the scan and especially if our scanning method were improved to allow for varying window size.

Although the starting and ending positions (on hg18) were listed in Table S3 for each of the 2040 candidate inversions, they can by no means be considered as the estimated breakpoints. Instead, these regions are just the windows within which PCA showed a structure of three equidistant clusters according to the criteria we set. All we can say about these windows is that there may be an inversion region that overlaps with each of the windows to such an extent that PCA can be used to identify it. To have a more accurate estimate on the locations of the predicted inversions, we could analyze the genotype frequencies of SNPs for the three inversion genotypes within and outside the window using the method shown in Figure S6. This procedure can only be performed manually one region at a time and better be conducted using a large sample. We therefore leave it to a future investigation. Nevertheless, the information given in Table S3 is sufficient for inversion-disease association studies in a genome-wide fashion, because the locations listed there can be used to infer inversion genotypes using SNP data.

**Figure 7:**
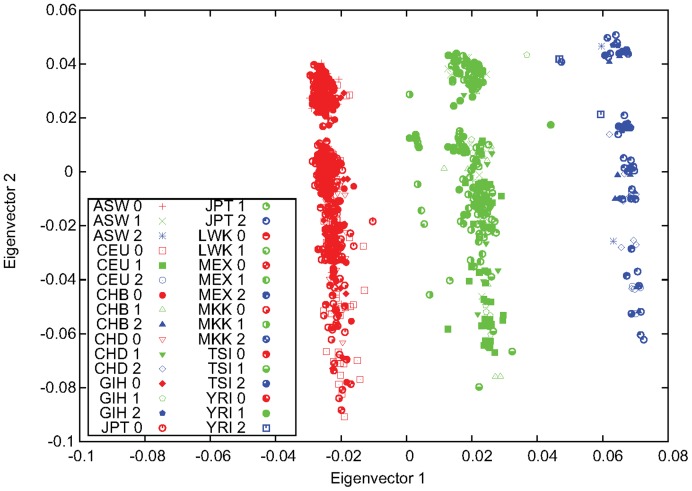
Genotyping of a predicted inversion at 3q21.3 for the HapMap populations. The first two eigenvectors obtained from PCA performed for all 11 HapMap populations using markers inside the predicted 3q21.3 inversion region.

An example of our predicted inversion polymorphisms is located at 3q21.3. In our scanning using PCA, the region was first identified as a window from 126441217 to 126902462 (hg18). By analyzing the allele frequencies of SNPs for the three groups of inversion genotypes, we obtained a more accurate estimate of the region: from 126426580 to 126585334 (hg18), as shown in Figure S6. The three-stripe pattern was clear for the combination of all HapMap populations, as shown in Figure 7. Here again, we supposed that the stripe with fewer samples and little variation represented the inverted homozygous individuals. Genotyping of this inversion polymorphism was then straightforward, and the results are listed in Table S1. Mendelian consistency was confirmed for all populations with family information, as shown in Table S2. The three-stripe pattern was clear for most of the single-population PCA (Figure S5). It is interesting to note that there was a within-stripe pattern for JPT, similar to that observed for CEU for the 8p23.1 and 17q21.31 inversions.

The genotype frequencies, allele frequencies, and the P values of the HWE tests for this predicted inversion for all populations are listed in Table 4. No deviation from HWE (P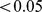) was observed for this predicted inversion. The Asian populations and MEX have a high frequency of inversion (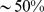), the African populations have a low frequency (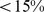), and the Caucasian populations (including GIH) have a frequency in between. This may indicates a geographical selection on this predicted inversion. The presence of this predicted inversion is not supported by the fosmid pair-end mapping conducted by [28] for the seven HapMap samples, three of whom were shown to be inverted heterozygous by the PCA method, as shown in (Table S1). Therefore, this may be a false positive discovery, and should be taken mainly as an illustrative example of how we can genotype an inversion even if the locations of the breakpoints are not determined and how we can narrow down their locations.

**Table 4:** Genotype and allele frequencies and the P values for HWE tests of the predicted inversion at 3q21.3.

	Homozygous		Homozygous	Inversion	Hardy-Weinberg
Population	non-inverted	Heterozygous	inverted	frequency	P values
ASW	0.68(48)	0.30(21)	0.03(2)	0.18	1.0000
CEU	0.49(80)	0.46(74)	0.05(8)	0.28	0.1154
CHB	0.26(21)	0.55(45)	0.20(16)	0.47	0.5051
CHD	0.29(20)	0.46(32)	0.26(18)	0.49	0.4792
GIH	0.52(43)	0.39(32)	0.10(8)	0.29	0.5951
JPT	0.26(21)	0.41(34)	0.33(27)	0.54	0.1270
LWK	0.90(75)	0.10(8)	0.00(0)	0.05	1.0000
MEX	0.24(17)	0.52(37)	0.24(17)	0.50	0.8149
MKK	0.80(137)	0.19(32)	0.01(2)	0.11	1.0000
TSI	0.57(44)	0.42(32)	0.01(1)	0.22	0.0992
YRI	0.75(123)	0.23(38)	0.01(2)	0.13	1.0000

## Discussion

A common practice in genome-wide association studies (GWAS) is to perform PCA using high-density genotype data of markers across the whole genome (or of a set of ancestral informative markers) to detect and correct for population stratification. Inside an inversion region, if recombination in inversion heterozygotes is suppressed, a special population substructure may be created without geographic isolation: two distinct groups of inversion homozygotes with different orientations, and their 1∶1 admixture consisting of the inversion heterozygotes. For simulated inversions and the well-known inversions in the HapMap data, we have demonstrated that locally performed PCA can readily detect this special substructure and thus can serve as a powerful and cost-effective tool to identify inversion polymorphisms and genotype samples that are polymorphic for an inversion. We have genotyped most of the samples of the 11 HapMap populations using PCA for the inversion regions, including the two well-known ones at 8p23.1 and 17q21.31, and have partially validated the obtained genotypes by checking Mendelian consistency using the family information of some of the HapMap populations. We have also conducted an autosomal genome-wide scan using PCA, and predicted 2040 inversion polymorphisms in the Caucasian populations, including 169 previously reported inversions listed in the DGV.

Compared with the sequencing-based or cytogenetic approaches, our PCA method has intrinsic limitations. First, detecting and genotyping inversions using PCA can be done only for a large group of samples with all three inversion genotypes and for relatively large inversions, because sample size and number of markers have a strong influence on the performance of PCA [23]. Second, when the distinction between different inversion orientations is not extremely sharp, individuals located between two stripes in the eigenvector space cannot be genotyped with high accuracy. To determine whether the individuals are located between two stripes simply by chance or because of a rarely occurring recombination between two inversion orientations, one has to resort to sequencing-based or cytogenetic approaches. Third, distinguishing between the two inversion homozygous groups using PCA by assuming that less variation exists among the inverted homozygotes is not always reliable, although this difficulty may be overcome by pooling the data of interest with those of samples with known characteristics of the inversion under consideration. Fourth, when used to detect novel inversions, the PCA-based method cannot accurately determine the locations of the breakpoints. Fifth, since our PCA approach is based on the assumption that recombination is suppressed between inverted and non-inverted chromosomal segments, false negatives may occur when suppression is only moderate and the initial inversion happened too long ago. The 8p23 inversion in the African populations and the Asian populations may be an example. False positives may also occur because suppression of recombination may be caused by other mechanisms than inversions. Sixth, there is evidence of recurrence [6] for some inversion polymorphisms. Like other SNP-based approaches, the PCA approach may not be suitable for genotyping or detecting such inversions, such as the 8p23 inversion in the Asian populations. Finally, if an inversion is relatively young, the two orientations are not distinct enough and a local substructure is not expected. Therefore, inversions of young ages can not be detected or genotyped using the PCA-based approach.

The advantages of our method are also obvious. Although the sequencing-based method using fosmid, clone-based analysis and the cytogenetic approach using FISH analysis are useful for detection and refinement of inversions, they are not effective for a large number of samples needed to characterize inversions in different populations. In another PCA approach used by Deng et al. [26], which is also based on high-density SNP data, inversion is detected and genotyped according to a two-cluster pattern in the scatter plot of the first few principal components. However, their method is based on phased genotype data, namely haplotype data, which are barely available on a genome-wide scale. In addition, our inversion diagnostic, namely the three-stripe pattern, should be more reliable than the two-cluster criterion in the haplotype-based PCA, which might have causes other than inversions. However, the advantage of the haplotype-based PCA approach is that it can be directly applied to detecting and genotyping inversion polymorphisms in the X chromosome. Our PCA-based approach in its present form cannot be applied to the X chromosome because different variances between males (with genotypes 0 and 1) and females (with genotypes 0, 1, and 2) will complicate the structure analysis. The statistical approaches proposed in [10], [11] is not powerful when the inversion frequency is not high enough (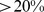), and cannot be used to genotype inversions. As GWAS is becoming successful and genome-wide high-density SNP genotype data are now abundant for various human populations, our PCA method, which is based on only unphased SNP genotype data, is not only a cost-efficient tool for characterizing important, known inversion polymorphisms in various human populations, but also potentially useful in detecting novel inversions in a genome-wide fashion, as demonstrated by our genome-wide scanning. Although it is difficult to estimate how many of those predicted inversions represent true inversions, our experience with the known inversions implies that further investigations, especially validation using the sequencing-based approach or FISH analysis, are desirable. Improving our genome-wide scanning algorithm would contribute to the construction of the map of inversions across the human genome and to use this map to explore the distributions of inversions in human populations and the role of inversions in recent primate evolution.

Ideally, our PCA-based method should be applied to homogeneous populations because signals of inversion may be confounded by population stratification. However, in practice, we may deliberately pool data from different populations either to improve power or to help to determine the inversion genotype of a non-polymorphic population of interest using samples from other populations with known inversion genotypes. This is possible only for populations that are genetically so close that in a small region of inversion population stratification is not a problem. For the inversions we studied here, the non-African populations can be pooled for this purpose, probably except for the 8p23 inversion. For the 17q21.31 inversion, all populations can be pooled together because the inversion-caused variation is much stronger than that caused by population stratification. Special attention should be paid to the situations when admixed samples are included in PCA together with samples from populations that are the parental populations or are genetically very close to the parental populations. The pattern of eigenvector-plot reflecting the true admixture has to be distinguished from that cause by inversion. The former can be observed in a region wide enough to include sufficient markers that are informative to the genetic distinction between the parental populations, whereas the latter can only be seen inside an inversion region or a region that is dominated by an inversion. In general, if the admixture history is not too long, the true admixture pattern cannot be seen inside a region as small as an inversion, because recombination is rare between chromosomal segments of different ancestry. For the inversion polymorphisms we have studied here, admixture did not cause a problem for the two admixed populations, ASW and MEX.

Like other structural variants, inversions are known to be associated with susceptibility to disease [1]–[3]. Our proposed approach can be easily applied to existing GWAS case-control data to test the association of given inversions with diseases. Prospectively, this approach may also be used to perform genome-wide association tests for inversions. Finally, findings in the present work may also have applications in SNP-disease association tests. The local substructures caused by inversion, as detected by our PCA approach, may pose false positives or reduce power in association studies of SNPs. This kind of local stratification can rarely be caught or corrected for in global, or genome-wide, PCA, unless the region of the structural variant is extremely long, such as the 8p23.1 inversion. Therefore, if some significant SNPs have been identified in a GWAS, we suggest that a local PCA should be performed in order to rule out the possibility of false-positive association caused by an inversion. However, if the presence of an inversion reduces the power to detect association between the disease and the SNPs within or near this region, we would not be able to find a signal. This might be one of the reasons why only a small portion of variation has been accounted for by SNPs discovered so far using GWAS.

## Methods

### Genotype Data

We used unphased genotype data from phase III of the International HapMap Project [21] consisting of 1115 individuals genotyped on 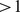 million SNPs. These individuals are from 11 different populations: 71 individuals of African ancestry in the Southwest United States (ASW); 162 Utah residents with Northern and Western European ancestry from the CEPH collection (CEU),; 82 Han Chinese in Beijing, China (CHB); 70 Chinese in Metropolitan Denver, Colorado (CHD); 83 Gujarati Indians in Houston, Texas (GIH); 82 Japanese in Tokyo, Japan (JPT); 83 Luhya in Webuye, Kenya (LWK); 71 individuals of Mexican ancestry in Los Angeles, California (MEX); 171 Maasai in Kinyawa, Kenya (MKK); 77 Tuscans in Italy (TSI); and 163 Yoruba in Ibadan, Nigeria (YRI). Among these 11 populations, there are 29 trios in ASW (20 trios have only one parent genotyped), 53 trios in CEU (5 trios have only one parent genotyped), 26 trios in MEX, 28 trios in MKK and 55 trios in YRI. This family information enabled us to check the Mendelian consistency of the inferred genotypes of the inversion polymorphisms studied here.

### Principal Components Analysis

PCA is now widely used as a tool for detecting population structures using high-density genotype data [23], [24], [29], [30]. The traditional form of PCA, in which the markers are treated as features, can be thought of as projecting the sampled individuals into a subspace spanned by the top principal components (PCs). Samples from the same population are found to form a cluster in this subspace, indicating that the top PCs reflect variations due to population structure in the sample. Here, following our previous work [23], we adopted another form of PCA, in which individuals, instead of markers, are treated as features. For detecting population structure, this form of PCA is equivalent to the traditional one, because the plot of the first few eigenvectors is equivalent to the PC-plot. We used this new form of PCA because the pattern of the plot in the space spanned by the first few eigenvectors (the eigenvector plot) can be directly related to the population parameters describing population differentiation [23]. Specifically, our formulation enabled us to theoretically justify and better understand the relationship between population structure with admixture and the pattern of the eigenvector plot. This form of PCA is thus suitable for our goal in this work, as inversion can be viewed as a special admixture between the inverted and non-inverted segments (see Results for more details). PCA was performed using R for markers inside individual inversion regions.

The K-means algorithm, as implemented in the R package *kmeans* [31], was used to assign an individual to one of the three clusters corresponding to the three inversion genotypes (see Results for more details) in the eigenvector plot following PCA. Because the sample sizes of the HapMap populations are small, the exact tests of HWE [32] were performed for an inversion using the estimated inversion genotypes for individual populations.

### Simulating Inversions

We used invertFREGENE [25] to simulate inversion events, which allowed us to assess the performance of our method of detecting inversion polymorphisms using PCA. invertFREGENE is a simulator of inversions in population genetic data using the forward-in-time algorithm and the assumption that recombination is suppressed between the inverted and non-inverted segments. A program called SAMPLE in the package can be used to sample genotype and haplotype data from the output of invertFREGENE simulations. Information on the inversion genotype of each individual can also be obtained from the output, enabling us to assess the performance of our inversion genotyping method using PCA.

### Scanning the HapMap Data for Inversion Polymorphisms

We performed a whole-genome scan for inversion polymorphisms by applying PCA within each window. To obtain a larger sample size, we performed a scan for the pooled data of the two Caucasian populations (CEU and TSI) because they are genetically similar. To make PCA meaningful, we should include a sufficient number of markers in a window. However, if the window size is too large, power to detect small- or medium sized inversions will be reduced. Based on our experience with known inversions, the window size was chosen such that 150 markers were included. The window slid forward by 10 markers each time. A window was picked up as a candidate inversion region if the plot of the first two eigenvectors showed a structure of three equidistant clusters (see Results). The K-means algorithm was used to determine if such a structure was obtained in a window: The within-cluster sum of squares (WSS) should be smaller than 0.08 and a parameter, 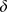, measuring the deviation of the middle cluster from the middle point of the two side clusters, should be smaller than 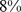. The parameter, 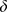, is defined as follows
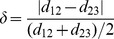
(2)where 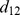 and 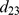 are the distances between the centroid of the middle cluster (2) and the centroids of the two side clusters (1 and 3), respectively. These two cutoffs were chosen based on our analysis of the known inversion at 8p23.1.

Unix shell scripts and programs written in C++ and R for genome-wide scan and inversion genotyping are available from the authors upon request.

### Web Resources

Database of Genomic Variants, http://projects.tcag.ca/variation/.

## Supporting Information

Figure S1 The first two eigenvectors obtained from PCA performed for each of the 11 HapMap populations using markers inside the 8p23.1 inversion region. The inversion genotypes were obtained by inspecting these single-population PCA results together with those from the PCA for pooled data shown in Figure 1 and Figures S2 and S3.
(PDF)

Figure S2 The first two eigenvectors obtained from PCA performed for pooled data of MEX and TSI with data of each of the other HapMap populations, represented by XXX, (except for CHB, for which the results are shown in Figure 4) using markers inside the 8p23.1 inversion region.
(PDF)

Figure S3 The first two eigenvectors obtained from PCA performed for pooled data of CEU, GIH, MEX, and TSI using markers inside the 8p23.1 inversion region. Genotyping of GIH was mainly based on this figure.
(PDF)

Figure S4 The first two eigenvectors obtained from PCA performed for each of the 11 HapMap populations using markers inside the 17q21.31 inversion region. The inversion genotypes were obtained by inspecting Figures 5 and 6.
(PDF)

Figure S5 The first two eigenvectors obtained from PCA performed for each of the 11 HapMap populations using markers inside the predicted 3q21.3 inversion region. The inversion genotypes were obtained by inspecting Figure 7.
(PDF)

Figure S6Estimation of the location of the predicted inversion at 3q21.3 by analyzing the marker allele frequencies. The inversion region was initially identified as a window from 12644127 to 126902462 in the genome-wide scan for inversion. The allele frequencies of SNPs around this region were then calculated in each of the three groups of HapMap samples with different inversion genotypes. Inside the inversion region, the allele frequency of the heterozygous group (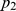) can be expressed in terms of those for the two homozygous groups (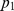 and 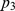) as follows: 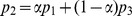 only when 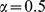. Outside the inversion region, the expression should be valid for any 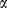 with 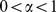. We therefore plotted the values of 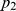, 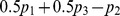, and 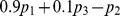, and estimated the inversion region as from 126426580 to 126585334, because inside this region the difference between 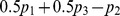 and 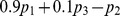 was significantly large.(PDF)

Table S1Inversion Genotypes of HapMap Populations for the Three Inversions Investigated (non-inverted homozygous: 0, inversted heterozygous: 1, inverted homozygous: 2. inversion status unknown: 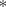).(XLSX)

Table S2 Mendelian Consistency of the HapMap Populations for the Three Inversions Investigated (missing: x, non-inverted homozygous: 0, inversted heterozygous: 1, inverted homozygous: 2.).
(XLSX)

Table S3 Candidate Inversion Polymorphisms Predicted by Genome-Wide Scan Using PCA (hg18).
(XLSX)
