# Supplementary material for: Investigation of Inversion Polymorphisms in the Human Genome Using Principal Components Analysis
Source: PLoS One. 2012 Jul 9;7(7):e40224. doi: 10.1371/journal.pone.0040224 (PMC3392271; doi:10.1371/journal.pone.0040224)
Supplement: Figure S1 — The first two eigenvectors obtained from PCA performed for each of the 11 HapMap populations using markers inside the 8p23.1 inversion region. The inversion genotypes were obtained by inspecting these single-population PCA results together with those from the PCA for pooled data shown in Figure 1 and Figures S2 and S3. (PDF) [file pone.0040224.s001.pdf]

Investigation of Inversion Polymorphisms in the Human Genome using Principal Components Analysis

Jianzhong Ma, Christopher I. Amos

Department of Genetics, The University of Texas MD Anderson Cancer Center, Houston, TX 77030, USA

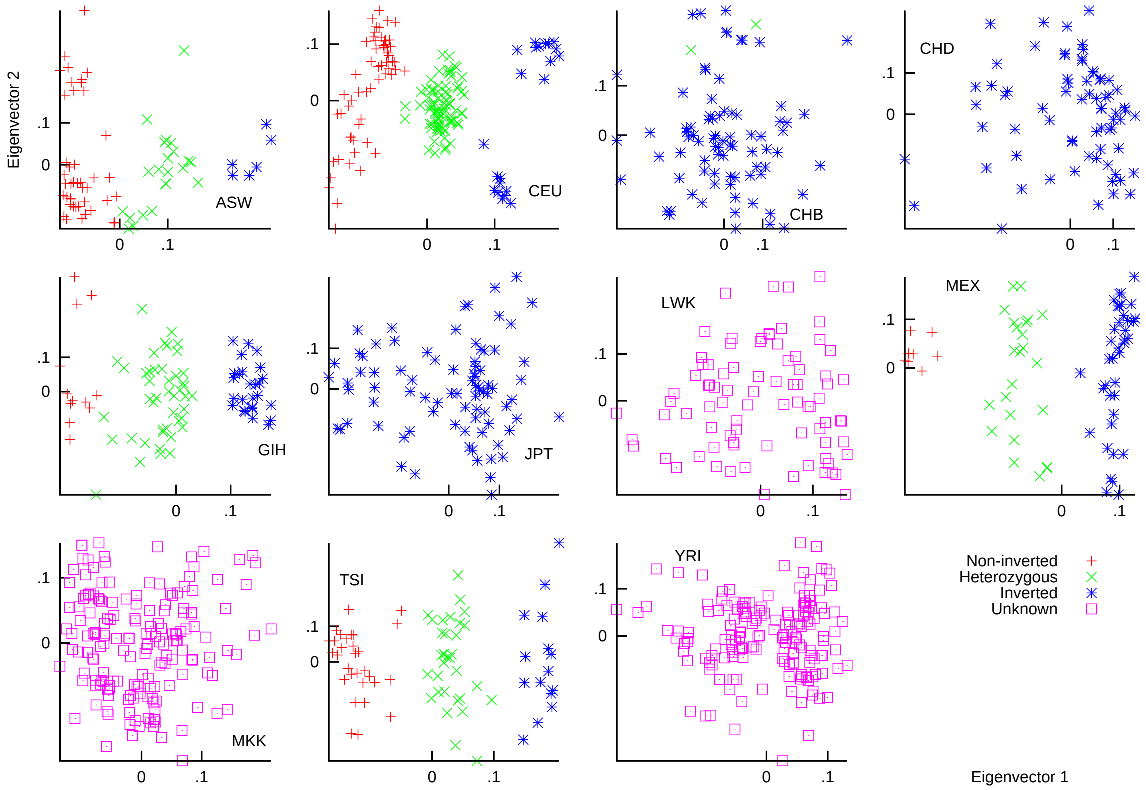

Figure S1. The first two eigenvectors obtained from PCA performed for each of the 11 HapMap populations using markers inside the 8p23.1 inversion region. The inversion genotypes were obtained by inspecting these single-population PCA results together with those from the PCA for pooled data shown in Figure 1 and Figures S2 and S3.
