# Supplementary material for: Investigation of Inversion Polymorphisms in the Human Genome Using Principal Components Analysis
Source: PLoS One. 2012 Jul 9;7(7):e40224. doi: 10.1371/journal.pone.0040224 (PMC3392271; doi:10.1371/journal.pone.0040224)
Supplement: Figure S2 — The first two eigenvectors obtained from PCA performed for pooled data of MEX and TSI with data of each of the other HapMap populations, represented by XXX, (except for CHB, for which the results are shown in Figure 4) using markers inside the 8p23.1 inversion region. (PDF) [file pone.0040224.s002.pdf]

Investigation of Inversion Polymorphisms in the Human Genome using Principal Components Analysis

Jianzhong Ma, Christopher I. Amos

Department of Genetics, The University of Texas MD Anderson Cancer Center, Houston, TX 77030, USA

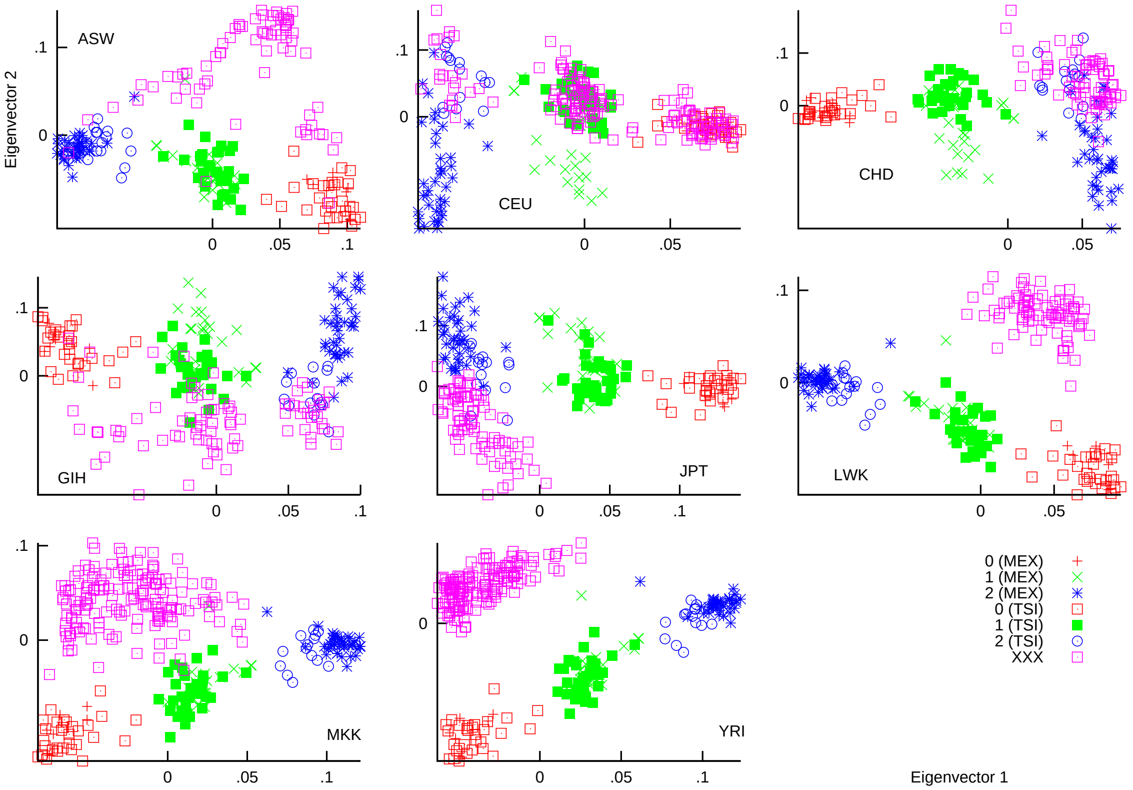

Figure S2. The first two eigenvectors obtained from PCA performed for pooled data of MEX and TSI with data of each of the other HapMap populations, represented by XXX, (except for CHB, for which the results are shown in Figure 4) using markers inside the 8p23.1 inversion region.
