# Supplementary material for: Investigation of Inversion Polymorphisms in the Human Genome Using Principal Components Analysis
Source: PLoS One. 2012 Jul 9;7(7):e40224. doi: 10.1371/journal.pone.0040224 (PMC3392271; doi:10.1371/journal.pone.0040224)
Supplement: Figure S3 — The first two eigenvectors obtained from PCA performed for pooled data of CEU, GIH, MEX, and TSI using markers inside the 8p23.1 inversion region. Genotyping of GIH was mainly based on this figure. (PDF) [file pone.0040224.s003.pdf]

Investigation of Inversion Polymorphisms in the Human Genome using Principal Components Analysis

Jianzhong Ma, Christopher I. Amos

Department of Genetics, The University of Texas MD Anderson Cancer Center, Houston, TX 77030, USA

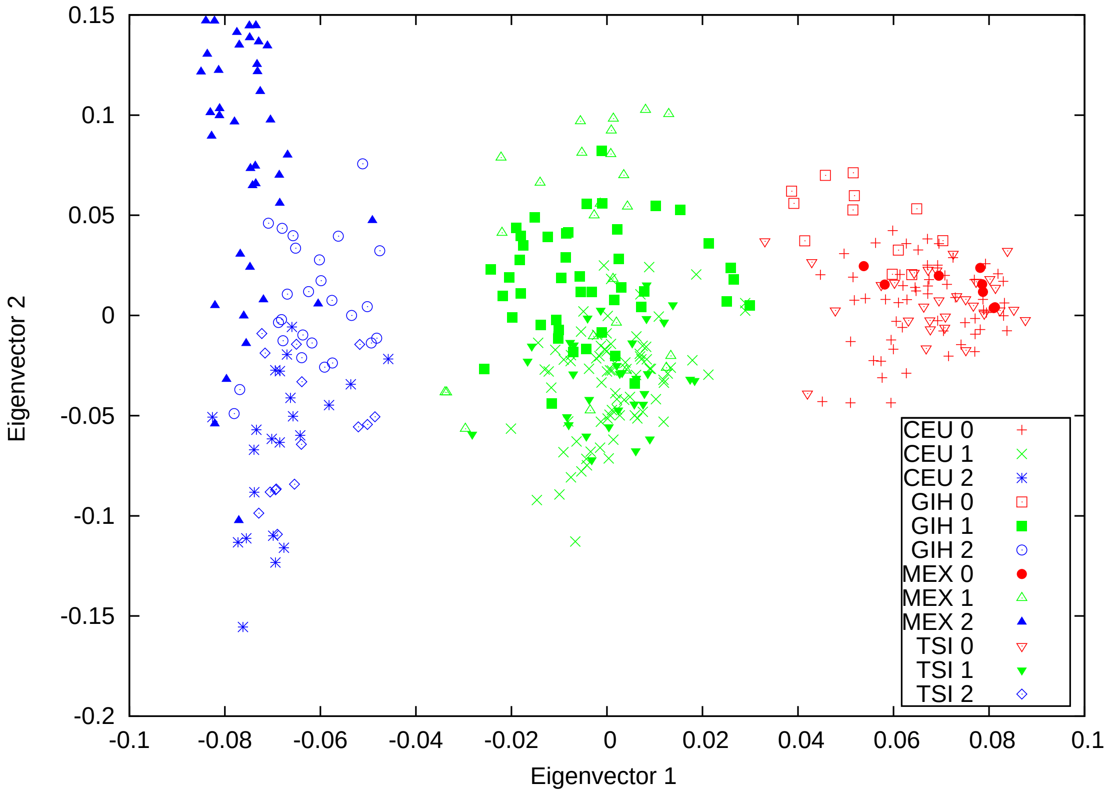

Figure S3. The first two eigenvectors obtained from PCA performed for pooled data of CEU, GIH, MEX, and TSI using markers inside the 8p23.1 inversion region. Genotyping of GIH was mainly based on this figure.
