# Supplementary material for: Investigation of Inversion Polymorphisms in the Human Genome Using Principal Components Analysis
Source: PLoS One. 2012 Jul 9;7(7):e40224. doi: 10.1371/journal.pone.0040224 (PMC3392271; doi:10.1371/journal.pone.0040224)
Supplement: Figure S4 — The first two eigenvectors obtained from PCA performed for each of the 11 HapMap populations using markers inside the 17q21.31 inversion region. The inversion genotypes were obtained by inspecting Figures 5 and 6. (PDF) [file pone.0040224.s004.pdf]

Investigation of Inversion Polymorphisms in the Human Genome using Principal Components Analysis

Jianzhong Ma, Christopher I. Amos

Department of Genetics, The University of Texas MD Anderson Cancer Center, Houston, TX 77030, USA

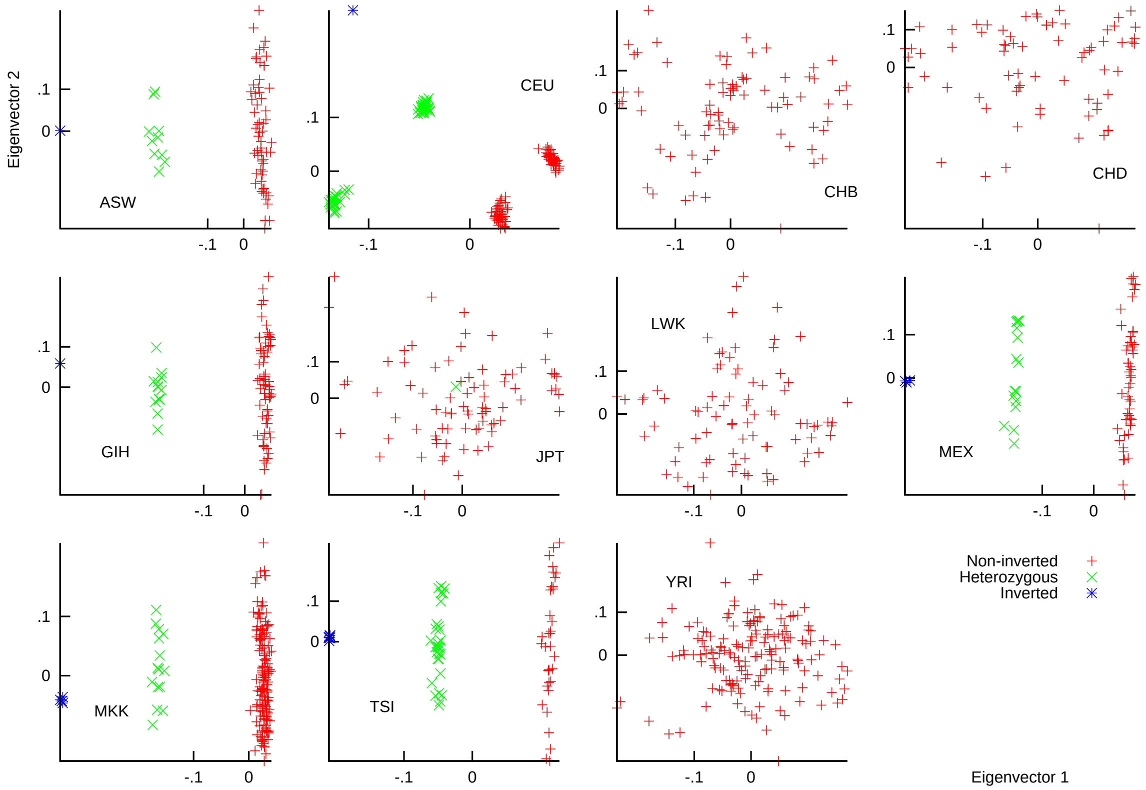

Figure S4. The first two eigenvectors obtained from PCA performed for each of the 11 HapMap populations using markers inside the 17q21.31 inversion region. The inversion genotypes were obtained by inspecting Figures 6 and7.
