# Supplementary material for: Investigation of Inversion Polymorphisms in the Human Genome Using Principal Components Analysis
Source: PLoS One. 2012 Jul 9;7(7):e40224. doi: 10.1371/journal.pone.0040224 (PMC3392271; doi:10.1371/journal.pone.0040224)
Supplement: Figure S5 — The first two eigenvectors obtained from PCA performed for each of the 11 HapMap populations using markers inside the predicted 3q21.3 inversion region. The inversion genotypes were obtained by inspecting Figure 7. (PDF) [file pone.0040224.s005.pdf]

Investigation of Inversion Polymorphisms in the Human Genome using Principal Components Analysis

Jianzhong Ma, Christopher I. Amos

Department of Genetics, The University of Texas MD Anderson Cancer Center, Houston, TX 77030, USA

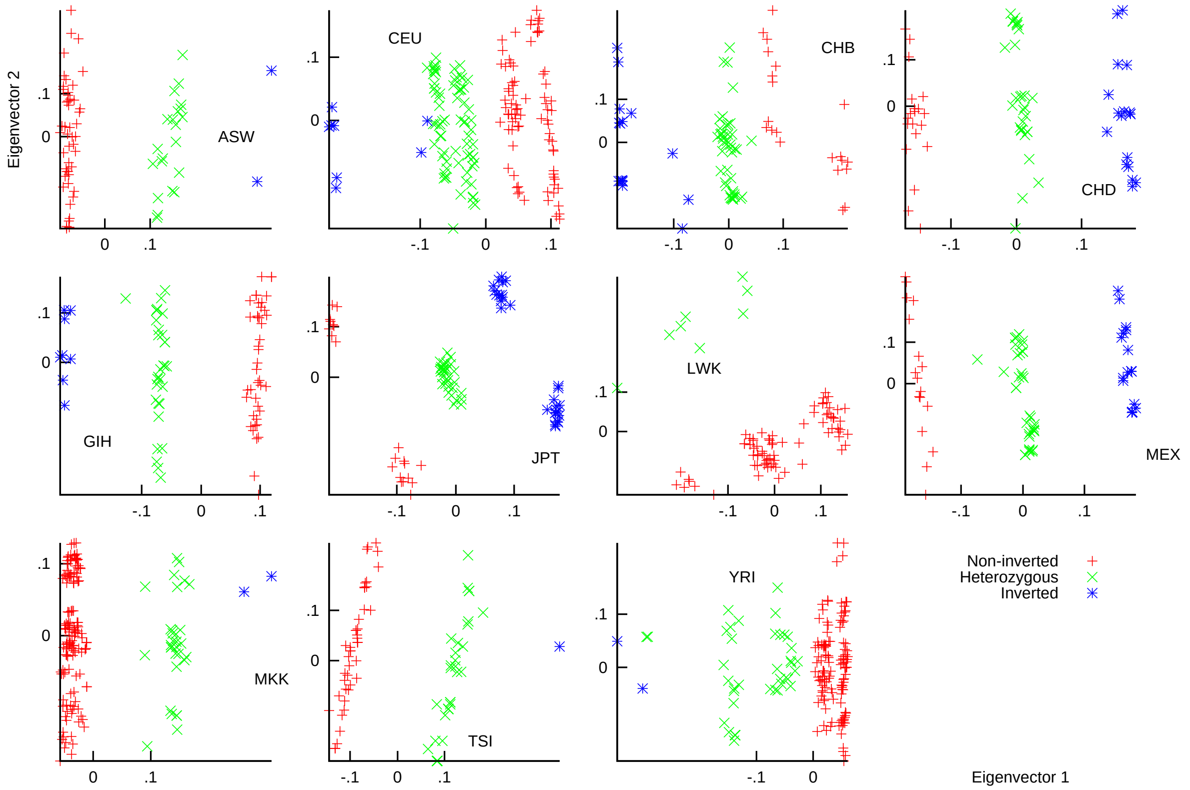

Figure S5. The first two eigenvectors obtained from PCA performed for each of the 11 HapMap populations using markers inside the predicted 3q21.3 inversion region. The inversion genotypes were obtained by inspecting Figure 9.
