# Supplementary material for: Investigation of Inversion Polymorphisms in the Human Genome Using Principal Components Analysis
Source: PLoS One. 2012 Jul 9;7(7):e40224. doi: 10.1371/journal.pone.0040224 (PMC3392271; doi:10.1371/journal.pone.0040224)
Supplement: Figure S6 — Estimation of the location of the predicted inversion at 3q21.3 by analyzing the marker allele frequencies. The inversion region was initially identified as a window from 12644127 to 126902462 in the genome-wide scan for inversion. The allele frequencies of SNPs around this region were then calculated in each of the three groups of HapMap samples with different inversion genotypes. Inside the inversion region, the allele frequency of the heterozygous group () can be expressed in terms of those for the two homozygous groups ( and ) as follows: only when . Outside the inversion region, the expression should be valid for any with . We therefore plotted the values of , , and , and estimated the inversion region as from 126426580 to 126585334, because inside this region the difference between and was significantly large. (PDF) [file pone.0040224.s006.pdf]

Investigation of Inversion Polymorphisms in the Human Genome using Principal Components Analysis

Jianzhong Ma, Christopher I. Amos

Department of Genetics, The University of Texas MD Anderson Cancer Center, Houston, TX 77030, USA

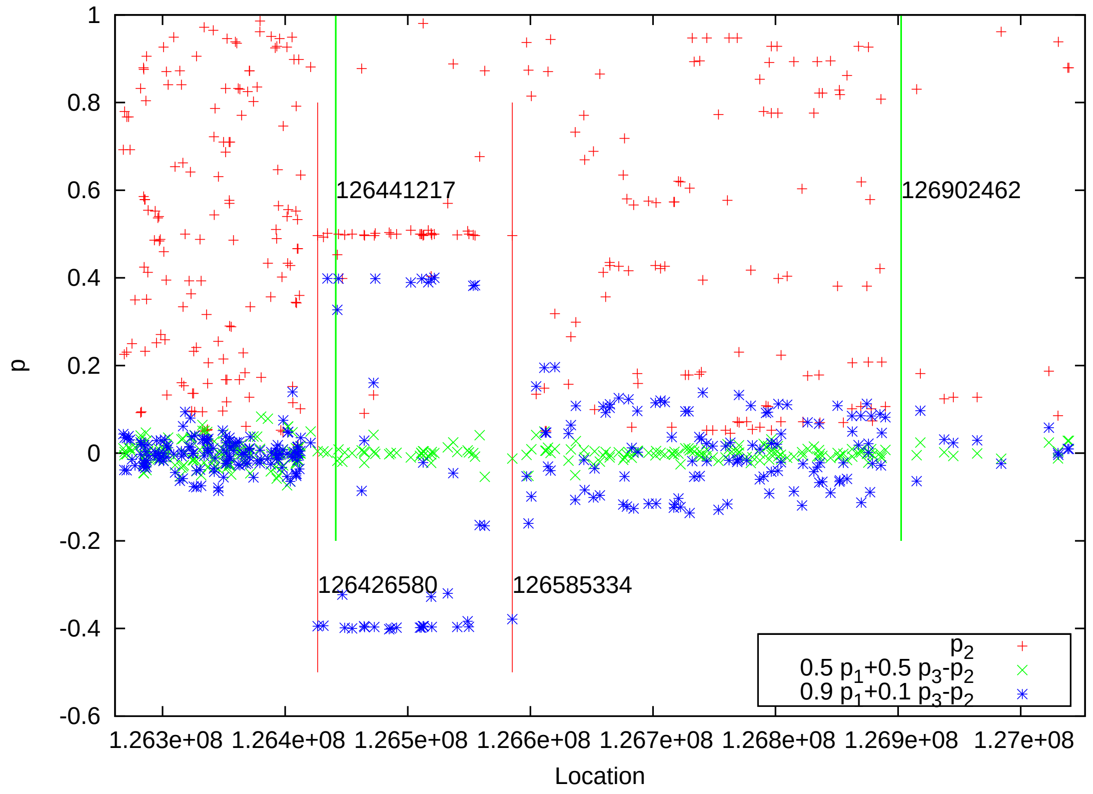

Figure S6. Estimation of the location of the predicted inversion at 3q21.3 by analyzing the marker allele frequencies. The inversion region was initially identified as a window from 12644127 to 126902462 in the genome-wide scan for inversion. The allele frequencies of SNPs around this region were then calculated in each of the three groups of HapMap samples with different inversion genotypes. Inside the inversion region, the allele frequency of the heterozygous group (p_2) can be expressed in terms of those for the two homozygous groups (p_1 and p_3) as follows: $p_2 = \alpha p_1 + (1-\alpha)p_3$ only when $\alpha = 0.5$. Outside the inversion region, the expression should be valid for any α with $0 < \alpha < 1$. We therefore plotted the values of p_2, $0.5p_1 + 0.5p_3 - p_2$, and $0.9p_1 + 0.1p_3 - p_2$, and estimated the inversion region as from 126426580 to 126585334, because inside this region the difference between $0.5p_1 + 0.5p_3 - p_2$ and $0.9p_1 + 0.1p_3 - p_2$ was significantly large.
